# Spontaneous Bone Regeneration (“Phoenix Jaw” Phenomenon) Following Segmental Mandibulectomy for Stage 3 Medication-Related Osteonecrosis of the Jaw: A Case Report

**DOI:** 10.7759/cureus.89370

**Published:** 2025-08-04

**Authors:** Kiyosato Hino, Shogo Kikuta, Jingo Kusukawa

**Affiliations:** 1 Dental and Oral Medicine, Kurume University School of Medicine, Kurume, JPN

**Keywords:** medication-related osteonecrosis of the jaw, mronj, phoenix jaw phenomenon, segmental mandibulectomy, spontaneous bone regeneration

## Abstract

Segmental mandibulectomy is a valid treatment for advanced medication-related osteonecrosis of the jaw (MRONJ), but subsequent reconstruction is challenging. Spontaneous bone regeneration (SBR) following resection is exceedingly rare, particularly in the elderly. We present the case of a 63-year-old woman with stage 3 MRONJ who underwent a 60 mm segmental mandibulectomy. The defect was reconstructed with a titanium plate, with meticulous preservation of the periosteum. Over a three-year follow-up period, serial imaging demonstrated progressive and substantial bone regeneration along the plate, resulting in a robust, continuous mandibular segment without any bone grafting. This case highlights that SBR, a phenomenon metaphorically termed the "Phoenix jaw," can occur even in older patients with advanced MRONJ, challenging conventional expectations regarding bone regeneration in this patient population.

## Introduction

Medication-related osteonecrosis of the jaw (MRONJ) is a refractory condition characterized by exposed necrotic bone in the maxillofacial region. This condition is a well-documented complication arising from the long-term administration of bone-modifying agents, including bisphosphonates and anti-Receptor Activator of Nuclear Factor-κB Ligand (RANKL) monoclonal antibodies. It has a debilitating impact on the quality of life of affected patients, particularly those with osteoporosis or underlying malignancies [1,2]. The American Association of Oral and Maxillofacial Surgeons' (AAOMS) staging system classifies the disease from Stage 0 to Stage 3 based on clinical signs and symptoms [1]. In osteoporotic patients, the risk of MRONJ is estimated to be 0.02-0.05% with bisphosphonates and 0.04-0.3% with denosumab. Its pathophysiology is understood to involve a complex interplay of anti-angiogenic and anti-resorptive effects, leading to impaired bone remodeling and increased susceptibility to infection. While conservative treatment may be appropriate for early-stage MRONJ, advanced stages frequently necessitate aggressive surgical intervention, such as segmental mandibulectomy, to control the disease process [3,4].

Spontaneous bone regeneration (SBR) following mandibular segmental resection remains an extremely rare phenomenon, despite accumulating evidence for the efficacy of such extensive surgical interventions for MRONJ [5]. Previous studies suggest that several factors may contribute to this phenomenon, including meticulous periosteal preservation, stabilization of bony segments, rigorous infection control, and inductive stimulation from surrounding soft tissues [6-9]. This periosteal-driven osteogenesis, occurring in the absence of grafting, is a phenomenon metaphorically termed the "Phoenix jaw." The term draws an analogy from the mythological phoenix, symbolizing rebirth from destruction, and reflects the unexpected reconstitution of mandibular bone following extensive necrosis and resection. It is important to note that "Phoenix jaw" serves as a descriptive analogy for SBR, rather than a standardized clinical entity. This phenomenon encompasses both spontaneous regeneration of necrotic jawbone after pathological fracture [10] and regeneration observed following extensive mandibular resection [11,12]. Herein, we represent a notable case of SBR following segmental mandibulectomy for stage 3 MRONJ and its reconstruction with a titanium plate.

## Case presentation

Written informed consent was obtained from the patient for the case report and the publication of images. The study was approved by the Ethics Committee of Kurume University School of Medicine (approval No. 24212) and was conducted in accordance with the Declaration of Helsinki.

A 63-year-old woman underwent extraction of the lower right third molar at a local dental clinic in January 2020. This was followed by a persistent, non-healing socket. Despite local interventions and systemic antibiotic therapy, her symptoms failed to resolve, and she subsequently developed paresthesia of the right lower lip and chin. She was referred to our center in May 2020 for the management of unremitting pain and bone exposure in the right mandible.

**Figure 1:**
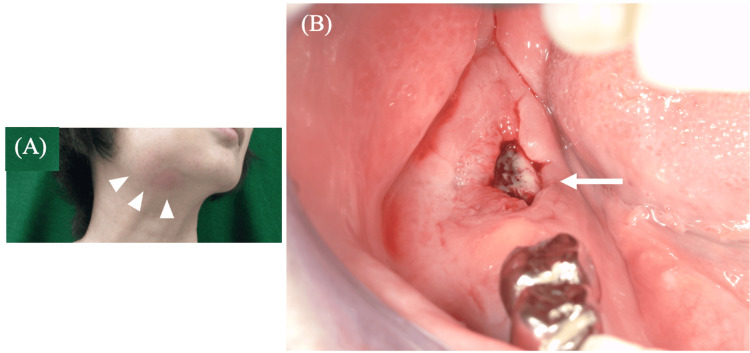
Initial clinical findings (A) Swelling with redness and warmth observed from the right mandible to the submandibular region (white arrowheads). (B) Mucosal defect and exposure of necrotic bone noted around the right second mandibular molar (white arrow).

Her medical history included left breast cancer (T4bN1M1, Stage IV) with bone metastasis. She had received intravenous zoledronic acid (a bisphosphonate) for 24 months from April 2018 to March 2020. Unfortunately, the specific dosage of zoledronic acid could not be retrieved from the patient's medical records. Initial clinical assessment revealed a prominent, indurated swelling, approximately 30 × 40 mm in size, spanning the right mandibular body and submandibular region (Figure 1A).

**Figure 2:**
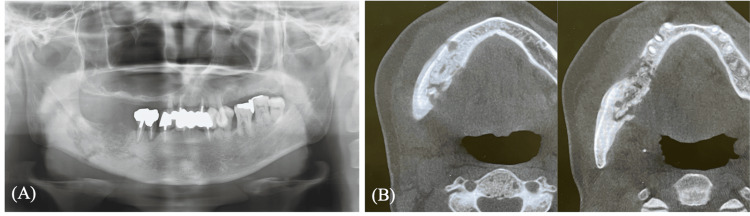
Preoperative radiographic findings (A) Panoramic radiograph showing bone resorption reaching the inferior border of the mandible; (B) Cone-beam Computed Tomography (CBCT) image reveals sclerotic changes extending from the right mandibular body to the anterior region of the mandibular angle.

Concurrently, paresthesia was confirmed in the right lower lip and mental region. The intraoral examination identified a 7 × 10 mm area of exposed, discolored necrotic bone in the region of the right mandibular second molar, which was enveloped by erythematous and diffusely swollen mucosa (Figure 1B). A panoramic radiograph demonstrated destructive osteolytic changes extending to the inferior border of the mandible (Figure 2A).

Cone-beam computed tomography (CBCT) further delineated extensive sclerotic changes from the right mandibular body to the angle (Figure 2B). The lesion was diagnosed as stage 3 MRONJ, according to the diagnostic criteria of the AAOMS, based on the presence of exposed bone with infection and extensive osteolysis extending to the inferior border of the mandible on radiographic imaging. Under general anesthesia, a segmental resection of the right mandible was performed. A full-thickness mucoperiosteal flap was carefully elevated with meticulous preservation of the native periosteum. Preoperatively, the area of osteonecrosis identified on radiographic imaging was used to estimate the extent of mandibular resection. Intraoperatively, the final resection margins were determined based on direct visual inspection. Areas showing discoloration were included as necrotic bone, while punctate bleeding from the bone surface was considered an indicator of viable bone, and served as the landmark for the resection endpoint. The resection length was 60 mm, and continuity was immediately restored using a 2.5 mm Matrix MANDIBLE Preformed Reconstruction Plate (DePuy Synthes, Raynham, MA, USA). The surgical procedure lasted approximately 4.5 hours, with an estimated blood loss of 80 mL. After segmental resection, the dead space was closed using a periosteal submerged suture, and the wound was completely closed with a tight mucosal suture. A negative pressure suction drain was placed for two days to prevent fluid accumulation, with a total drainage volume of approximately 60 mL.

**Figure 3:**
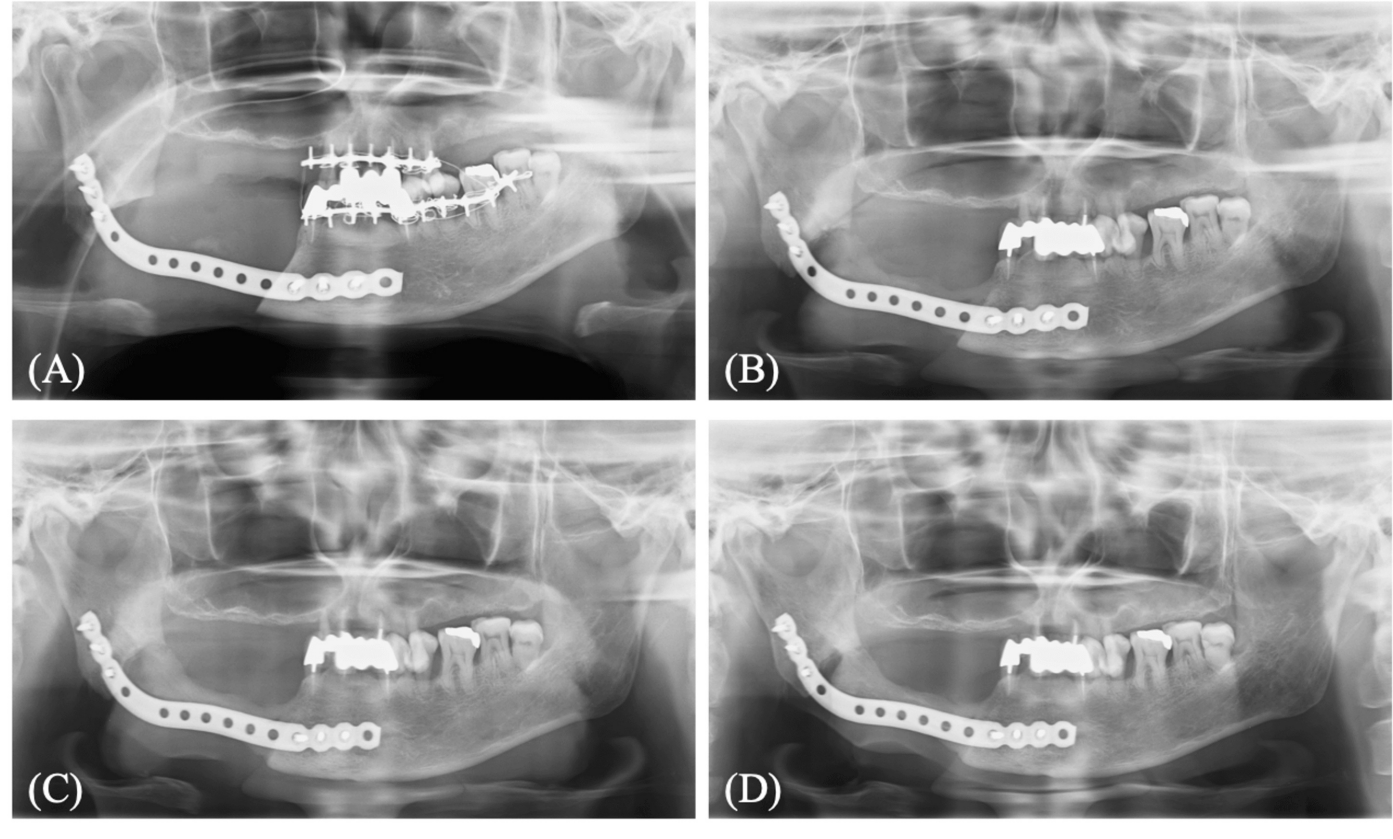
Postoperative panoramic radiographs Panoramic radiographs taken (A) immediately after surgery, (B) at one year, (C) 1.5 years, and (D) three years postoperatively. Progressive bone regeneration between the mandibular stumps was observed over time.

Intravenous antibiotics were administered for three days post-surgery. Oral intake was initiated on postoperative day seven. However, postoperative trismus was observed, necessitating the initiation of jaw-opening exercises from postoperative day seven. The patient was discharged on postoperative day 25, with no signs of infection or hardware-related complications. At the one-year postoperative follow-up, panoramic radiographs revealed a continuous bridge of newly formed bone along the reconstruction plate. This regeneration progressed over time, and by the three-year mark, the radiograph showed a robust, regenerated segment with a width comparable to the native mandible, albeit with a slightly reduced height (Figure 3).

**Figure 4:**
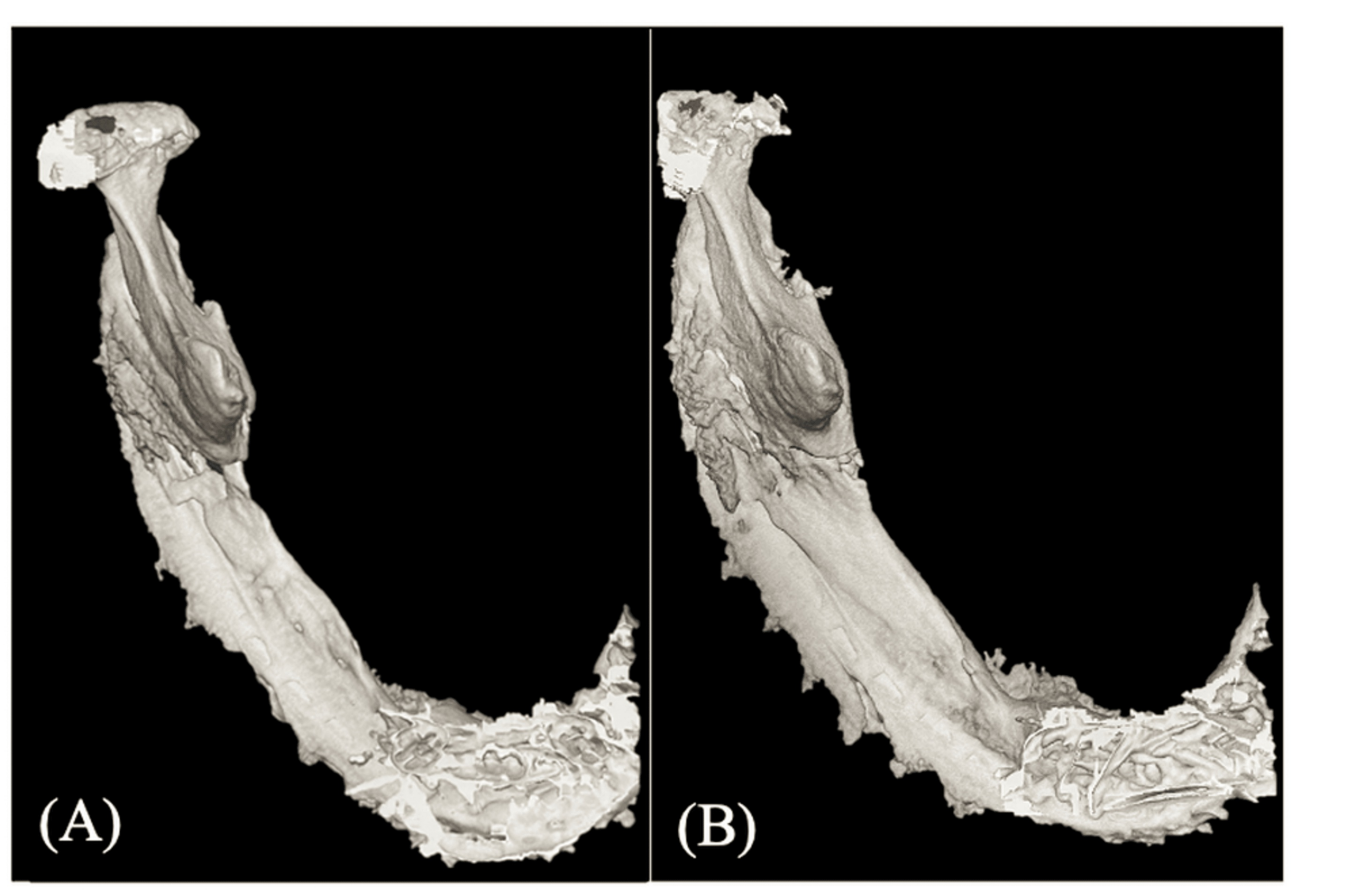
Postoperative cone-beam computed tomography (CBCT) images showing the progression of bone regeneration (A) At six months postoperatively, newly formed bone bridging the resection margins was observed. (B) At three years postoperatively, a continuous regenerated bone structure with substantial width was seen along the inner aspect of the reconstruction plate.

Serial CBCT imaging provided a clearer view of this progression: nascent bone formation was evident spanning the defect at just six months post-surgery, which matured into a well-defined, corticated structure with substantial width along the internal aspect of the plate by three years (Figure 4). 

To quantify this progression, serial measurements of the newly formed bone were taken at the midpoint of the resected area on CT scans. The bone width increased progressively from 6 mm at seven months to 12 mm at 21 months, after which it reached a plateau. Similarly, the bone height increased from 5 mm at seven months to 10 mm at 34 months and remained stable thereafter. 

To date, the patient remains asymptomatic with no clinical or radiographic evidence of MRONJ recurrence.

## Discussion

MRONJ is a debilitating condition characterized by exposed necrotic bone in the maxillofacial region, often associated with infection, pain, and impaired oral function. In advanced cases, extensive surgical resection is required [1]. While surgical resection is a validated treatment for stage 3 MRONJ [3], the resulting defects typically require complex reconstruction with bone grafts [2,5]. However, this is often not feasible due to the poor general condition of many MRONJ patients, for whom plate-only reconstruction remains the most viable, albeit functionally limited, option [5].

Remarkably, the present case demonstrates that substantial SBR, also referred to as the “Phoenix jaw” phenomenon, can occur following segmental mandibulectomy for advanced MRONJ. This finding is particularly noteworthy given that such regenerative capacity is generally considered limited in the elderly and bone metabolically altered by antiresorptive therapy [9-11]. SBR has traditionally been regarded as a phenomenon primarily seen in younger individuals, typically occurring after resections for benign tumors [6]. In general, the majority of MRONJ cases requiring segmental mandibulectomy do not exhibit SBR. However, a total of 21 cases, including the present one, have been reported in the literature in which SBR was observed following such resections. Notably, these cases occurred in a significantly older cohort (mean age: 71.9 years) [4,9,12-15] (Table 1), contradicting the prevailing assumption that SBR is limited to younger individuals or resections performed for benign lesions [6].

**Table 1:** Reported cases of spontaneous bone regeneration after segmental mandibulectomy for MRONJ, including the present case M: Male; F: Female; MRONJ: Medication-related osteonecrosis of the jaw. In the CAT classification system, C = condylar head, A = mandibular angle, and T = mental tubercle. TT refers to defects involving both mental tubercles. Combinations (e.g., AT or CAT) indicate which landmarks are involved. “Neck,” “body,” and “unclassified” refer to defects not involving these points.

Author	Publication year	Sex	Age	Treatment (CAT classification)	Immediate reconstruction	Medication history	Medication duration	Periosteum preservation	Time to bone formation	Extent of bone regeneration
Wilde et al. [12]	2011	M	55	TT	Reconstruction plate	Zoledronic acid	3 years	Yes	1 year	Continuous
Yazan et al. [13]	2016	M	72	body	No	Zoledronic acid	2 years	Yes	2 years	Continuous
Shinpei et al. [14]	2020	F	70	TAC	No	Zoledronic acid	ー	Yes	2 years	Continuous
Romanos et al. [9]	2020	F	67	TT	Reconstruction plate	Zoledronic acid	3 years	Yes	2 months	Continuous
	2020	F	70	T	No	Zoledronic acid	2 years	ー	<4 years	Continuous
Esen et al. [15]	2021	F	73	body	Reconstruction plate	Zoledronic acid	10 years	Yes	1 year	Continuous
Kwon et al. [4]	2024	F	73	body	Reconstruction plate	Bisphosphonates and denosumab	>4 years	Yes	<1 year	Continuous
	2024	F	73	body	Reconstruction plate	Bisphosphonates	>4 years	Yes	<1 year	Continuous
	2024	M	80	ー	Reconstruction plate	Bisphosphonates	2 years	Yes	Unknown	Partial
	2024	F	70	body	Reconstruction plate	Bisphosphonates	3 years	Yes	<1 year	Continuous
	2024	F	71	ー	Reconstruction plate	Bisphosphonates	3 years	Yes	<1 year	Continuous
	2024	F	85	ー	Reconstruction plate	Bisphosphonates	>4 years	Yes	Unknown	Partial
	2024	F	83	ー	Reconstruction plate	Bisphosphonates	>4 years	Yes	Unknown	Partial
	2024	F	78	ー	Reconstruction plate	Bisphosphonates	2 years	Yes	Unknown	Partial
	2024	F	71	TT	Reconstruction plate	Bisphosphonates	>4 years	Yes	<1 year	Continuous
	2024	F	50	T	Reconstruction plate	Bisphosphonates	3 years	Yes	<1 year	Continuous
	2024	M	62	ー	Reconstruction plate	Denosumab	>4 years	Yes	<1 year	Continuous
	2024	F	78	ー	Reconstruction plate	Bisphosphonates	1 year	Yes	<1 year	Continuous
	2024	F	87	ー	Reconstruction plate	Bisphosphonates	1 year	Yes	<1 year	Continuous
	2024	F	79	ー	Reconstruction plate	Denosumab	2 year	Yes	<1 year	Continuous
Present case	2025	F	63	body	Reconstruction plate	Zoledronic acid	22 months	Yes	1 year	Continuous

This apparent paradox suggests that the specific surgical conditions achievable during MRONJ treatment may create a uniquely pro-regenerative environment, even in older patients. SBR is considered multifactorial. Key contributing factors identified in previous reports include meticulous periosteal preservation [6], stabilization of the bony segments [7], rigorous infection control, and adequate vascular supply from surrounding soft tissues [5].

Anatomically, while SBR has been reported at various mandibular sites, the mandibular body is the most frequently observed location for regeneration [8,16]. Defect size is also a critical determinant; segments shorter than 12.6 cm are more favorable for regeneration in humans [6]. In the context of MRONJ, an analysis of 21 reported SBR cases shows that bisphosphonates were the predominant causative agent (18 cases), followed by anti-RANKL monoclonal antibodies (two cases). Although no significant differences in regenerative capacity based on drug type have been observed to date, the relationship between pharmacological parameters, such as drug type, administration duration, and withdrawal period, and SBR warrants further investigation [3]. The preservation of the periosteum is widely considered the most critical factor for enabling SBR [17]. Its indispensable role stems from multiple biological functions. The periosteum contains an inner cambium layer, rich in osteoprogenitor cells and periosteal stem cells, which provides critical resources for both intramembranous and endochondral ossification and secretes essential growth factors like Bone Morphogenetic Protein-2 (BMP-2) and Vascular Endothelial Growth Factor (VEGF) [18]. Additionally, it serves as a crucial physical barrier, preventing the infiltration of non-osteogenic granulation tissue and epithelium, thereby maintaining a secluded and favorable microenvironment for bone regeneration [9]. This meticulous preservation is a distinct advantage in MRONJ surgery compared to resections for malignancies, where the periosteum is often sacrificed to ensure adequate oncologic margins.

While high osteogenic potential is typically associated with younger patients [7,17], the cohort of 21 reported MRONJ cases with SBR demonstrates that this phenomenon can be achieved in elderly patients, provided the appropriate surgical conditions are met. Among these conditions, the role of rigid fixation is debated. Although the stability afforded by a reconstruction plate is thought to maintain the osteogenic space and provide a stable scaffold, SBR has also been documented in cases without plates [11,13,14]. This suggests that other factors, such as the micromechanical stimuli from oral functions, may also contribute by activating osteoblasts at the bone stumps and in the preserved periosteum. The regenerative timeline appears consistent across cases, with initial bone formation typically visible within the first year, followed by a maturation process that can extend beyond two years before reaching a structural plateau [3]. Our case aligns with this timeline, showing nascent bone on CBCT at six months and a well-defined structure by one year.

In the present case, the successful outcome was likely attributable to the confluence of these positive factors: meticulous periosteal preservation, stable fixation with a reconstruction plate, and sustained oral function. Nevertheless, SBR remains a largely unpredictable event. Clinicians should therefore avoid excessive reliance on its occurrence and must manage patient expectations accordingly. While SBR presents a compelling, less morbid alternative to extensive grafting, more reliable reconstructive options should be prioritized for cases requiring predictable occlusal restoration for implant therapy.

## Conclusions

This case presents a rare instance of marked SBR following segmental mandibulectomy for MRONJ, representing the so-called “Phoenix jaw” phenomenon, characterized by periosteal-driven bone formation. Multiple factors are thought to be involved in the bone regeneration process, including meticulous periosteal preservation, stable internal fixation with a reconstruction plate, and stringent infection control. While this phenomenon offers a compelling alternative to more morbid reconstructive procedures, its unpredictability means it cannot yet be considered a standard primary treatment. Nevertheless, this case underscores the profound intrinsic regenerative capacity of the mandible, even under metabolically compromised conditions, and warrants further prospective studies to better define the surgical and biological prerequisites for this favorable outcome.
